# Implementation of a longitudinal, near-peer ECG didactic curriculum in an internal medicine residency program and impact on ECG interpretation skills

**DOI:** 10.1186/s12909-023-04483-y

**Published:** 2023-07-24

**Authors:** Matthew G. Kaye, Hassan A. Khan, Gregory D. Gudleski, Yan Yatsynovich, Susan P. Graham, Alysia V. Kwiatkowski

**Affiliations:** 1 University at Buffalo, State University of New York, Buffalo, NY USA; 2 Cookville Regional Medical Center, Cookville, TN USA; 3 Jacobs School of Medicine and Biomedical Sciences, Buffalo, NY USA

**Keywords:** Electrocardiography, Curriculum, Internship and residency, Education, Medical, Graduate

## Abstract

**Background:**

To determine the impact of a longitudinal, near-peer, dedicated ECG didactic curriculum on internal medicine resident ability to accurately interpret ECGs.

**Methods:**

This study employs a prospective cohort design. Internal medicine residents at University at Buffalo participated in monthly ECG didactic sessions over a 7-month period. Residents were invited to complete pre- and post-curriculum questionnaires. Responses were anonymous and participation voluntary. Data collected included basic demographics, career interest, exposure to clinical cardiology, and number of sessions attended. Residents were asked to interpret sixteen unique ECGs, divided evenly among eight common rhythms into both questionnaires. Pre- and post-curriculum cohorts were compared using t-tests and chi-square analyses. Associations between attendance, comfort level in interpretation, and number of correct interpretations were analyzed using Pearson correlations. Multivariate linear regression determined the strongest predictor of the number of correct ECG interpretations.

**Results:**

The post-curriculum cohort correctly interpreted a significantly greater percentage of ECGs compared to pre-curriculum cohort (74.5% vs. 60.9%, *p* < .001). Didactic attendance was significantly associated with comfort level in interpreting ECGs (*r* = .328, *p* = .018) and trended towards an increased number of correct interpretations (*r* = .248, *p* = .077). Residents who attended three or more sessions demonstrated increased ECG interpretation skills compared to those who attended two or fewer sessions (80.0% vs. 71.1%, *p* = .048). Number of clinical cardiology rotations attended was significantly associated with correct interpretations (r = .310, *p* < .001) and was the strongest predictor of accurately interpreting ECGs (β = 0.29, *p* = .037).

**Conclusions:**

Participation in a longitudinal, near-peer ECG didactic curriculum improved resident ability to interpret ECGs. A curriculum which contains both didactic sessions and clinical exposure may offer the greatest benefit in improving ECG interpretation skills.

## Background

The electrocardiogram (ECG) is one of the most commonly employed diagnostic tests in medicine. ECGs offer a simple, non-invasive, and cost-effective method for the early detection and diagnosis of cardiovascular disease [1, 2]. However, their clinical utility depends upon accurate interpretation. It has been reported that up to one-third of all ECG interpretations contain important errors [3]. Misinterpretation of ECGs is associated with poor patient outcomes and inferior clinical decision-making [4, 5].

Residents are often responsible for initial interpretation of ECGs and for implementing appropriate treatment decisions based on their findings. Although many medical school curricula include an introduction to ECGs, residents enter their training with varying competency in ECG interpretation [6, 7]. The American College of Physicians recommends that physicians receive training in ECG interpretation during their residencies [8]. Likewise, the American College of Cardiology and the American Heart Association have issued joint, evidence-based guidelines suggesting that ECG education involve bedside and clinical teaching [9]. The Accreditation Council for Graduate Medical Education has also issued recommendations that internal medicine residents be taught and attain competency in ECG interpretation [10].

Despite the recommendations from these professional societies, there exists no consensus on the best methods for teaching ECG interpretation to residents [11]. Commonly employed educational methods include lecture-based, workshop-based, self-directed, and web-based formats [12]. One educational model which has become increasingly popular in medical education is near-peer teaching. The near-peer teaching model establishes an educational relationship between learners and lecturers of similar learner level in which the lecturers are more advanced within the field of interest than the learners [13]. Near-peer teaching improves learner satisfaction and knowledge acquisition and has been shown to lead to superior ECG interpretation skills when compared to self-directed learning [14, 15]. Near-peers often have a better understanding of learners’ meta-cognitive needs, which may explain the effectiveness of near-peer teaching models [16].

We recently implemented a cardiology fellow-led ECG didactic curriculum for the Internal Medicine Residency Training Program at The University at Buffalo (State University of New York). This study builds upon our prior research demonstrating improvement in resident comfort-level in interpreting ECGs following this curriculum [17]. Herein, we aim to expand upon these findings to determine whether residents demonstrate objective improvement in ECG interpretation skills following participation in this year-long didactic curriculum. We further aim to determine whether certain resident characteristics, including specialty of interest and exposure to clinical cardiology, may serve as predictors of successful improvement in ECG interpretation skills.

## Methods

### ECG didactic curriculum

The University at Buffalo’s Internal Medicine Residency Training Program’s near-peer, didactic ECG curriculum aims to educate internal medicine residents on proper ECG interpretation, basic electrophysiologic pathology, and initial medical treatments for common arrhythmias. This curriculum was designed in conjunction with the Cardiovascular Disease Fellowship Program at The University at Buffalo and was led by cardiology fellows with cardiology attending oversight. Overall, eight cardiology fellows voluntarily participated in delivering ECG didactic sessions. Fellows who elected to participate in didactic sessions were assigned specific ECG-related content to address by the chief cardiology fellow. Didactic sessions were held in monthly intervals. Residents attended a total of seven didactic sessions over the course of seven months. Each didactic session ran for thirty minutes. Residents spent a total of three and a half hours on ECG training of the course of the seven months. Traditional lecture-style format was used to deliver background material on cardiac electrical abnormalities and to educate in the basics of interpreting various arrhythmias. Didactic sessions were held using a hybrid model of in-person and virtual teaching. Residents attended sessions at three training sites in-person, with videoconferencing used to connect residents across the different sites. The fellow leading each session would present virtually to all sites simultaneously. Approximately five to seven unique ECGs were discussed during each didactic lesson. Question-and-answer style format was employed at the end of each session to assess learner comprehension of the aforementioned material. Monthly didactic sessions were divided into the following broad topics: (1) Normal Cardiac Electrophysiology; (2) Basics of ECG Interpretation; (3) Acute Coronary Syndromes; (4) Heart Blocks and Conduction Disease; (5) Bundle Branch Blocks; (6) Wide-complex Arrhythmias; (7) Narrow-Complex Arrhythmias, (8) Miscellaneous ECG Abnormalities (including Brugada Syndrome, Long-QT Syndrome); (9) Basics of Pacemakers and Paced Rhythm Identification.

### Data collection

Data was collected using paper questionnaires. All residents who attended ECG didactic sessions were invited to complete two questionnaires administered before and after completion of the ECG curriculum. Participation in both questionnaires was voluntary and data collected anonymously. Data collected included demographics, attendance, exposure to clinical cardiology, interest in cardiology, prior participation in ECG training (if any), and number of didactic sessions attended. Residents were asked to eight common ECG arrhythmias and/or abnormalities on each questionnaire for a total of sixteen ECGs. Two unique ECGs were selected for each rhythm and divided evenly among the two questionnaires. Answers were given in free-text response and were scored for correct or incorrect identification of the rhythm or abnormality. Comfort-level in interpreting ECGs was assessed using a Likert scale. A separate survey was sent to the cardiovascular disease fellows to determine how participation in organizing and leading ECG didactic session affected their training and skills as educators.

### Statistical analysis

Descriptive analyses (means, SDs, %) were used to summarize characteristics of the sample and survey data. Correct ECG interpretations were compared between the pre- and post-curriculum cohorts using chi-square analyses for individual ECG rhythm strips and a t-test for the total number of correct interpretations. Associations between sample characteristics, survey responses and number of correct interpretations were analyzed using Pearson correlations for linear variables and chi-square analyses for categorical variables. Finally, a multivariate linear regression was used to determine the significant predictors of the number of correct ECG interpretations.

## Results

Characteristics of the residents who completed the surveys are shown in Table 1. There were no significant differences in the composition of Post-Graduate Year (PGY) levels for residents who participated in the pre- and post-curricular surveys. Overall, 17% of residents reported an interest in pursuing a career in cardiology, and there was no difference in post-graduate career interests between the groups. Residents attended an average of just over two ECG didactic sessions and 16.7% of the cohort attended four or more.

**Table 1:** Baseline characteristics of ECG lecture series participants

Characteristic	Pre-Curriculum (n = 68)	Post-Curriculum (n = 52)	*p*-value
PGY Level			0.131
1	39 (57.4%)	21 (40.4%)	
2	18 (26.5%)	16 (30.8%)	
3	11 (16.2%)	15 (28.8%)	
Has Interest in Cardiology	10 (14.7%)	10 (19.2%)	0.510
Number ECG Didactics Attended	N/A	2.11 (1.51)	N/A

PGY, post-graduate year; ECG, electrocardiogram

Residents reported feeling overall more comfortable interpreting ECGs at the end of the curriculum compared to pre-curriculum [3.12 (0.70) vs. 2.84 (0.81), *p* = .05, *d* = 0.37]. As PGY level increased, there was a statistically significant trend towards increased self-reported comfort in ECG interpretation abilities (Table 2).

**Table 2:** Comparisons of correct ECG interpretations for all residents by PGY level

	PGY Level			
	1 (n = 60)	2 (n = 34)	3/4 (n = 26)^§^	p-value
STEMI	50 (83.3%)	30 (88.2%)	20 (76.9%)	0.507
Atrial fibrillation	41 (68.3%)	19 (55.9%)	20 (76.9%)	0.214
2nd degree AV-Block Type 2	20 (33.3%)	15 (44.1%)	14 (53.8%)	0.185
Supraventricular tachycardia	28 (46.7%)	21 (61.8%)	16 (61.5%)	0.257
Ventricular tachycardia	54 (90.0%)	30 (88.2%)	25 (96.2%)	0.546
Atrial flutter	55 (91.7%)	29 (85.3%)	24 (92.3%)	0.556
2nd degree AV-Block Type 1	29 (48.3%)	17 (50.0%)	16 (61.5%)	0.517
3rd degree AV-Block	26 (43.3%)	20 (58.8%)	16 (61.5%)	0.184
Total correct (M, SD)	5.05 (1.59)	5.32 (1.43)	5.81 (1.58)	0.115
Comfort interpreting (M, SD)	2.75 (0.77)^a^	2.88 (0.74)^a^	3.54 (0.51)^b^	< 0.001**

^a^ and ^b^ superscripts are significantly different at p < .05; ^§^combined PGY-3s & 4s

** p ≤ .001

PGY, post-graduate year; STEMI, ST-elevation myocardial infarction; AV, atrioventricular

**Fig. 1:**
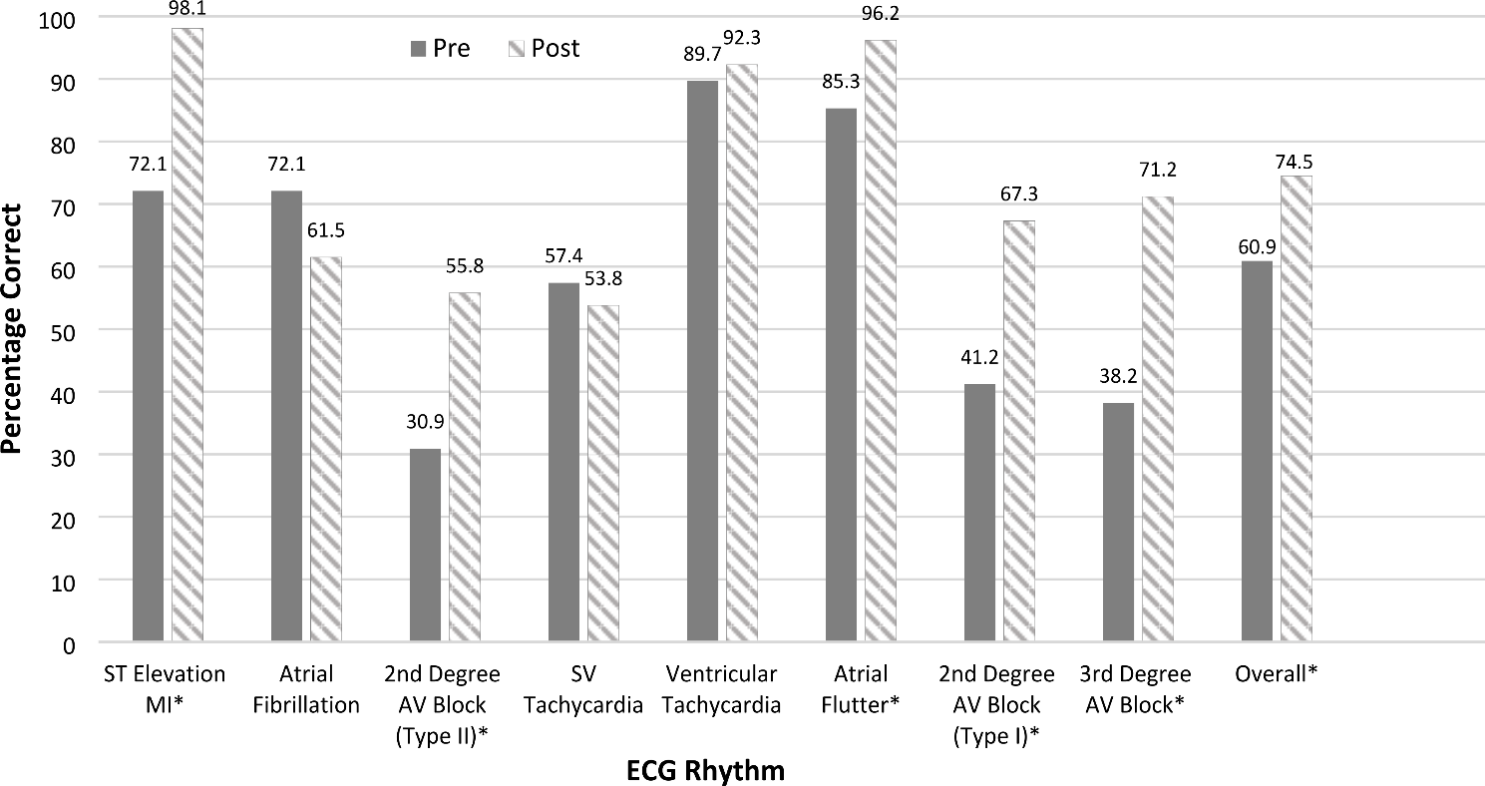
Differences in the percentage of correct ECG interpretations at pre-curriculum vs. post-curriculum * *p* < .05; MI = myocardial infarction; AV = atrioventricular; SV = supraventricular

Overall, the average number of correct ECG interpretations was significantly higher for the post-curriculum cohort than for the pre-curriculum cohort [5.96 (1.27) vs. 4.87 (1.55), respectively; *p* < .001, d = 0.76]. Regarding the individual ECG rhythms, the post-curriculum cohort had significantly higher percentages of correct identification of abnormalities than the pre-curriculum cohort for ST-elevation myocardial infarction (STEMI, *p* < .001), 2nd -degree AV block Mobitz type I (*p* = .005) and 2nd -degree AV block Mobitz type II (*p* = .006), 3rd degree AV block (*p* < .001), and atrial flutter (p = .049; see Table 3; Fig. 1). The pre-curriculum cohort was more successful at correctly identifying atrial fibrillation than the post-curriculum cohort (72.1% vs. 61.5%), although the difference was not statistically significant (*p* = .223).

**Table 3:** Comparisons of pre- and post-curriculum correct ECG interpretations for all residents

	Pre-curriculum (n = 68)	Post-curriculum (n = 52)	p-value
STEMI	49 (72.1%)	51 (98.1%)	< 0.001**
Afib	49 (72.1%)	31 (59.6%)	0.152
2nd degree AV-Block Type 2	21 (30.9%)	28 (53.8%)	0.011*
Supraventricular tachycardia	39 (57.4%)	26 (50.0%)	0.423
Ventricular tachycardia	61 (89.7%)	48 (92.3%)	0.625
Atrial flutter	58 (85.3%)	50 (96.2%)	0.049*
2nd degree AV-Block Type 1	28 (41.2%)	34 (65.4%)	0.009*
3rd degree AV-Block	26 (38.2%)	36 (69.2%)	< 0.001**
Total (M, SD)	4.87 (1.55)	5.85 (1.39)	< 0.001**
Comfort interpreting (M, SD)	2.84 (0.81)	3.12 (0.70)	0.050*

*Note*: ^a^ and ^b^ superscripts are significantly different at p < .05; ^§^combined PGY-3s & 4s

* p ≤ .05

** p ≤ .001

PGY, post-graduate year; STEMI, ST-elevation myocardial infarction; AV, atrioventricular

A statistically significant association was found between the total number of correct ECG interpretations and number of clinical cardiology rotations attended (*r* = .310, *p* < .001). This was the only variable among all (i.e., both pre- and post-curriculum) residents that showed a significant relationship with the number of correct ECG interpretations. For the post-curriculum residents only, there was a trend toward significance between the number of near-peer didactic sessions attended and the total number of correct interpretations (*r* = .248, *p* = .077). Further analysis revealed that residents who attended three or more didactic sessions had a significantly higher average number of correct ECG interpretations than those who attended two or fewer sessions [6.40 (1.23) vs. 5.69 (1.24); *p* = .048, *d* = 0.58]. Results of a multivariate regression showed that number of clinical cardiology rotations attended was a better predictor of accurate ECG interpretations than didactic sessions attended for the post-curriculum cohort (β = 0.293, *p* = .037, see Table 4).

**Table 4:** Regression analysis predicting accurate interpretation of ECGs

Variable	*B*	*SE*	β	*t*	*p*-value	95% CI
Didactic sessions attended	0.145	0.115	0.173	1.26	0.213	[-0.08–0.38]
Cardiology blocks taken	0.523	0.244	0.293	2.14	0.037*	[0.03–1.01]

* p < .05

The cardiology fellows who led the ECG didactic curriculum were surveyed and asked to reflect on their experience. Of the respondents, 85.7% either agreed or strongly agreed that they learned something during this experience and were better able to interpret the arrhythmias presented as a result of helping to facilitate the didactic sessions. All (100%) of the fellows who were surveyed indicated that they enjoyed the experience and agreed or strongly agreed with the statement that participation in the ECG didactic curriculum improved their skills as an educator (28.6% “Agree”, 71.4% “Strongly Agree”).

## Discussion

Results from our study affirmed that residents who attended the longitudinal, near-peer ECG didactic curriculum demonstrated improved accuracy in ECG interpretation. The impact of the didactic sessions themselves was evidenced by regression analysis, as individuals who attended more sessions were more likely to correctly interpret ECGs than their peers who attended fewer sessions. Residents also reported feeling more comfortable interpreting ECG tracings after attending didactics. Additionally, residents further along in their training reported feeling significantly more comfortable interpreting ECGs compared to their junior peers, although this did not translate into a significant difference in objective interpretations among residents of different training levels.

A unique finding of this study was the positive effect that participation in clinical cardiology had on ECG interpretation skills. While prior research has demonstrated that patient exposure improves overall clinical performance among healthcare workers, the present study, to our knowledge, is the first to specifically identify the impact of exposure specifically to clinical cardiology on ability to interpret ECGs [18]. Interestingly, there was no significant effect of interest in a post-residency career in cardiology on ability to accurately interpret ECGs, which suggests minimal to no confounding influence of this variable and that the potential for self-studying ECGs among residents interested in cardiology compared to those without an interest in cardiology did not factor into the results. These results suggest that a longitudinal curriculum which combines both didactic sessions and clinical exposure offers the greatest benefit in improving resident ECG interpretation skills.

ECGs demonstrating 2nd - and 3rd -degree AV blocks proved the most difficult for residents to accurately interpret prior to the implementation of the didactic curriculum. Previous research has corroborated the difficulty residents often have in correctly detecting these arrhythmias on ECG tracings [19, 20]. Another ECG abnormality which has often proved difficult for residents to accurately identify is evidence of posterior myocardial infarction [19, 21]. Although our present study did not specifically test for accuracy in interpreting localization of myocardial infarction, the cohort demonstrated significant improvement in generally detecting STEMI on ECG tracings after completion of the curriculum. This finding has potentially important clinical impact, as an abnormal ECG is often the first sign of an acute STEMI, and prior studies have shown that misdiagnosis or delayed diagnosis results in poorer outcomes and increased mortality [4].

Finally, results from our survey of the cardiology fellows who organized and facilitated the ECG didactic sessions indicated that their participation in the curriculum improved their own abilities to interpret the various arrhythmias presented and improved their abilities as educators. These results are in line with those reported by prior research, which has likewise found that near-peer teaching provides mutual benefit to both learners and educators and allows educators to consolidate their own knowledge [14], In future studies, we hope to employ more frequent surveying of the fellows regarding their experiences and to determine the effect of delivering ECG didactics on fellow ECG interpretation skills. This may include working more closely with fellowship program leadership and assigning didactic sessions to fellows in training based on their deficiencies to improve performance in these domains.

There are limitations in the present study. Our relatively small size and its single-center nature reduce generalizability. Given that data was collected via voluntary questionnaires, our study is subject to potential nonresponse bias, although demographic characteristics of residents who participated in both pre- and post-curricular surveys were similar. Although number of didactic sessions attended predicted increased resident aptitude at correctly identifying ECG abnormalities, the observational nature of our study limits our ability to establish a direct causal relationship.

Results of this study confirm the effectiveness of near-peer ECG didactic curriculum in improving resident ability to accurately interpret ECGs. While prior research has shown a similar effect in a single didactic session, to our knowledge, this is the first study to evaluate the impact of near-peer teaching on resident ECG interpretation skills in the context of a formal, longitudinal curriculum led by cardiovascular disease fellows [22, 23]. Further research would benefit from direct comparison of the effect of near-peer learning vs. a non-near-peer model within a longitudinal curriculum on resident acquisition of ECG interpretation skills.

## Conclusion

The implementation of a novel, near-peer, ECG didactic curriculum improved internal medicine resident ability to accurately interpret ECGs. The most significant improvement was seen in correct identification of STEMI, 2nd - and 3rd -degree AV block, and atrial flutter. Exposure to clinical cardiology in the form of dedicated clinical rotations was the strongest predictor of successful ECG interpretation. An ECG curriculum which contains both near-peer didactic sessions as well as clinical exposure to cardiovascular diseases may offer the greatest benefit in improving ECG interpretation skills.

## Data Availability

All data generated or analyzed during this study are available upon reasonable request. Please contact corresponding author Matthew G. Kaye, MD for all data inquiries.

## Abbreviations

ECG: Electrocardiogram
PGY: Post-Graduate Year
STEMI: Post-Graduate Year
STEMI: ST-elevation myocardial infarction
